# The Validity of the Push Band 2.0 during Vertical Jump Performance

**DOI:** 10.3390/sports6040140

**Published:** 2018-11-05

**Authors:** Jason P. Lake, Simon Augustus, Kieran Austin, Peter Mundy, John J. McMahon, Paul Comfort, Guy G. Haff

**Affiliations:** 1 Chichester Institute of Sport, University of Chichester, Chichester PO19 6PE, UK; s.augustus@chi.ac.uk (S.A.); kaustin2@stu.chi.ac.uk (K.A.); 2 Centre for Sport, Exercise and Life Sciences, Coventry University, Coventry CV1 5FB, UK; ab9674@coventry.ac.uk; 3 Directorate of Sport, Exercise and Physiotherapy, University of Salford, Frederick Road, Salford M6 6PU, UK; j.j.mcmahon@salford.ac.uk (J.J.M.); p.comfort@salford.ac.uk (P.C.); 4 Centre for Exercise and Sports Science Research, Edith Cowan University, Joondalup, Western Australia WA 6027, Australia; g.haff@ecu.edu.au

**Keywords:** method comparison, field testing, jump testing, neuromuscular function, athlete monitoring, accelerometer

## Abstract

The Push Band has the potential to provide a cheap and practical method of measuring velocity and power during countermovement vertical jumping (CMJ). However, very little is known about whether it conforms to laboratory-based gold standards. The aim of this study was to assess the agreement between peak and mean velocity and power obtained from the belt-worn Push Band, and derived from three-dimensional motion capture, and vertical force from an in-ground force platform. Twenty-two volunteers performed 3 CMJ on a force platform, while a belt-worn Push Band and a motion capture system (a marker affixed to the Push Band) simultaneously recorded data that enabled peak and mean velocity and power to be calculated and then compared using ordinary least products regression. While the Push Band is reliable, it tends to overestimate peak (9–17%) and mean (24–27%) velocity, and when compared to force plate-derived peak and mean power, it tends to underestimate (40–45%) and demonstrates fixed and proportional bias. This suggests that while the Push Band may provide a useful method for measuring peak and mean velocity during the CMJ, researchers and practitioners should be mindful of its tendency to systematically overestimate and that its measures of peak and mean power should not be used.

## 1. Introduction

Countermovement vertical jump (CMJ) testing using a force platform is now routinely conducted across a variety of sporting domains, as well as in many sports science research studies [1]. This is because changes in CMJ strategy (i.e., the underpinning force and time characteristics before take-off) that either maintain or change jump height (JH) between testing occasions are thought to provide insight into neuromuscular function and fatigue [2,3]. However, the cost and the availability of other potentially more practical methods of assessing CMJ performance provide strength and conditioning practitioners with options to assess CMJ related parameters [4]. One such method is the Push Band. This is an accelerometer-based system that can be worn either in a sleeve on the forearm or barbell, or on a waist belt. However, very little is known about how the waist-borne method compares to laboratory-based gold standard methods of assessing parameters like peak and mean velocity and peak and mean power.

Only five studies to date, to the authors’ knowledge, have attempted to establish the validity of performance metrics produced by the Push Band [4,5,6,7,8]. Two of the studies utilized the back-squat exercise [5,6], one utilized the bicep curl and shoulder press exercises [7], and two utilized the CMJ [4,8]. Unfortunately, the studies to date that have attempted to establish the validity of the Push Band for quantifying CMJ parameters have only been published in abstract form [4,8], and thus full details of these studies’ methodologies are unknown. From the available information, it is understood that Montalvo et al. [4] reported a moderate correlation (*r* = 0.641) between a Push Band (placed on the participants’ waist) and force platform (criterion method). Unfortunately, these authors did not state which independent variable was used in their correlational analysis (presumably it was jump height), while correlational analysis does not assess agreement [9]. Ripley and McMahon [8] reported a strong correlation for both peak velocity (*r* = 0.918) and peak power (*r* = 0.949), during the CMJ, between the Push Band (placed on the participants’ forearm) and force platform. However, the Push Band significantly (*p* < 0.001) overestimated velocity and power when compared to the force platform.

Recently, Push released their 2.0 system, and to the authors’ knowledge, this system has not been validated during CMJ. This therefore represents a gap in the literature that, if filled, could provide strength and conditioning practitioners with data to help inform them about whether this system agrees with laboratory-based gold standards. Therefore, the aim of this study was to assess agreement between peak and mean velocity and power obtained from the belt-worn Push Band, and derived from three-dimensional motion capture, and vertical force from an in-ground force platform. Based on literature that has assessed the validity of the Push Band during jumping and different resistance exercises [4,5,6,7,8], it was hypothesized that the Push Band would demonstrate poor agreement with laboratory-based gold standard methods.

## 2. Materials and Methods

### 2.1. Subjects

Twenty-two healthy individuals (18 men, 4 women, age: 22.5 ± 5.3 years, body mass: 81.5 ± 13.3 kg, height: 1.75 ± 0.07 m) who regularly participated in a variety of university-level sports (e.g., soccer, rugby (i.e., league and union), basketball and volleyball), volunteered to participate in this study and provided written informed consent. Subjects were excluded if they had suffered from a lower-body injury in the 6 months leading up to data collection. The study was approved by the institutional ethics committee and conformed to the principles of the World Medical Association’s Declaration of Helsinki.

### 2.2. Procedures

**Figure 1:**
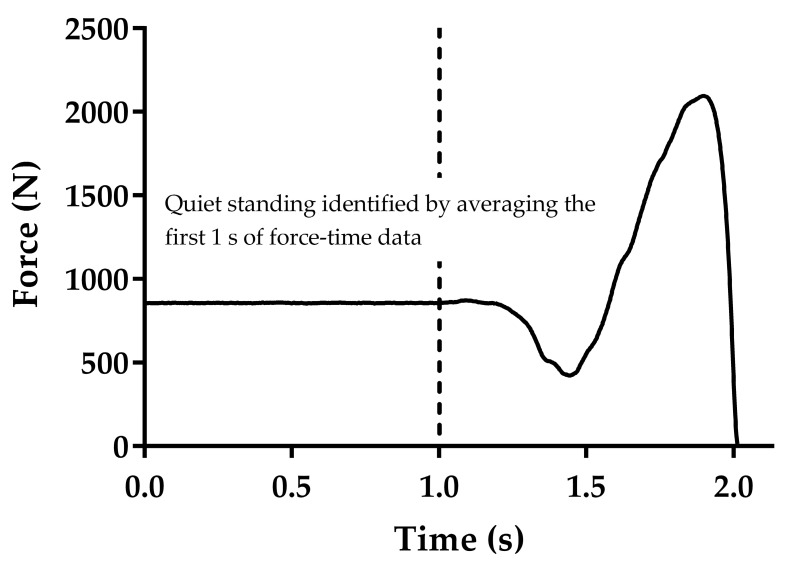
The identification of quiet standing force from vertical force-time data.

All subjects performed a standardized dynamic warm-up before all testing. This began with 2–3 min of upper- and lower-body dynamic stretching using a previously described warm up [10]. Specifically, subjects performed 2 circuits of 10 repetitions each of ‘arm swings’, ‘lunge walk’, ‘walking knee lift’, and ‘heel to toe lift’ [11], and unloaded, sub-maximal CMJs. Subjects then performed three bilateral CMJ interspersed with 60 s of rest [12]. To remove the impact of arm movement, subjects kept their hands on their hips throughout each jump. Before jump initiation subjects placed their hands on their hips and positioned each foot centrally on the force platform. Subjects were then instructed to stand perfectly still until given the words of command: “stand by, go!” The first word of command was issued 2 s after the instruction to stand perfectly still and indicated the start of data acquisition [12]; this 2 s gap was to ensure that a sufficient period of quiet standing (Figure 1) was recorded [12,13]. Subjects were instructed to jump “as fast and as high as possible”. Jump performances were watched to ensure that subjects kept their hands on their hips throughout each jump. Trials were repeated if these criteria were not met.

### 2.3. Data Collection

All jumps were captured concurrently using the Push Band 2.0 (Push Inc., Toronto, ON, Canada) recording at 200 Hz, one force platform (Kistler Type 9287C, Kistler Instruments, Hampshire, UK) that recorded vertical force at 1000 Hz, and a 10-camera, opto-electronic 3D motion analysis system (Vicon T40S, Vicon Motion Systems, Oxford, UK), sampling at 200 Hz. The Push Band was set to jump-mode and attached to the waist belt supplied with the system as per manufacturer recommendations. Mean and peak vertical velocity and power values from the propulsion phase of each jump were sent via Bluetooth to an Apple iPhone 6 running the proprietary Push application (V4.2.1). A single reflective marker (12.6 mm diameter) was attached to the Push Band directly superior to the center of the sensor. The motion capture system recorded the three-dimensional displacements of the marker during each repetition in Vicon Nexus software (V2.6, Vicon Motion Systems, Oxford, UK).

### 2.4. Data Analysis

Data were calculated from the three trials and then the trial with the highest peak velocity (center of mass velocity from the force data) was selected for further analysis and validity was assessed using data from the different methods from this trial. Additionally, to assess within-session reliability, we used these data and the data from the trial with the second-highest peak velocity (center of mass from the force data). We used this approach because this is considered the gold standard way of measuring center of mass velocity during vertical jumping, and because vertical jump height is underpinned by vertical take-off velocity and peak velocity typically provides a very similar value. The trials that the highest and second-highest peak velocity (center of mass from the force data) occurred in were identified on a subject-by-subject basis, and corresponding data from the other variables and methods of interest were taken from this trial. Raw force data were analyzed using a customizable spreadsheet following the methods recently described and used by Lake et al. [14]. Velocity was obtained by integrating acceleration with respect to time using the trapezoid rule using the method described by Owen et al. [13]. Acceleration was obtained by dividing the net vertical force by body mass. Briefly, body weight was obtained by averaging one second of force-time data as the participants stood still (quiet standing) while awaiting the word of command to jump. This was recorded during each trial, and the subject was instructed to stand perfectly still. The standard deviation (SD) of the quiet standing phase was calculated, and the start threshold of body weight ±5 standard deviations was calculated. The final part of this process was to then go back through the force-time data by 30 ms, as it has been shown that this positions the start at a point when the subject is still motionless. Therefore, the assumption of zero velocity was not negatively compromised, which could impact the calculation of subsequent kinetic and kinematic data [13,14].

Marker displacement data were exported to Visual 3D (V6.01.22, C-Motion, Rockville, MD, USA), where they were filtered using a fourth-order, zero-lag, Butterworth low-pass filter with cut-off frequency of 12 Hz. Data were visually inspected to assess the effect that different cut-off frequencies (6–20 Hz) had on vertical velocity and 12 Hz was selected because lower cut-off frequencies attenuated peak values. Motion capture velocity and acceleration were obtained using the finite difference method in Visual 3D. Motion capture power was calculated by first calculating the force applied to jumper, the body mass represented by the marker affixed to the Push Band; this was then multiplied by marker velocity. Force was calculated using the following equation:

Force = (body mass × acceleration of gravity) + (body mass × marker acceleration)

The propulsion phase was identified as the period between the first post countermovement positive velocity (center of mass velocity from the force method) and take-off. Take-off (for both motion capture and force methods) was identified in the three stages recently described and used by Lake et al. [14]. First, the first post-countermovement force value less than 10 N and the next force value greater than 10 N were identified; second, points 30 ms after and before these points, respectively, were identified to determine the center ‘flight phase’ array; third, mean and SD ‘flight phase’ force was calculated, and mean ‘flight phase’ force plus 5 SD was used to identify take-off [14]. We chose the 5 SD threshold because it provides the most robust approach, with the chance of it not identifying a ‘real’ change in force being 1 in 3.5 million [15]. The propulsion phase was automatically identified for the Push Band method using its proprietary software. 

### 2.5. Statistical Analyses

Numerous tests have been proposed as appropriate for establishing the reliability and validity of measurements within sports science [9,16,17,18]. Although no consensus exists about the most appropriate test, there are several limitations with the more commonly used tests (e.g., correlation, ordinary least-squares regression) [9,16,17,18]). It is outside the scope of this article to discuss each of these limitations, particularly as they have been discussed extensively elsewhere (Batterham and George [19], Ludbrook [16,17] and Mullineaux et al. [18]). Briefly, it has been stated that the principal limitation of the majority of the more commonly used tests is that they do not consider both fixed and proportional biases [18]. As such, it is suggested that comparative studies should use ordinary least-products regression [18], which considers both of these elements. However, because other assessments of reliability and validity (standard error of the mean (SEM), coefficient of variation (CV), intraclass correlation (ICC, *r*)) are routinely used [19], we have also included these methods for parity.

Before assessing reliability and validity, normality, uniform distribution and linearity were assessed. Ordinary least-products regression was used to determine fixed and proportional bias between data from the motion capture, force platform and Push Band using the methods described by Ludbrook [16,17]. If the 95% confidence interval for the intercept (x) did not include 0, then fixed bias was present. If the 95% confidence interval for the slope (y) did not include 1.0, then proportional bias was present. The strength of the ICC (<0.1 = trivial, 0.1–0.3 = small, 0.3–0.5 = moderate, 0.5–0.7 = high, 0.7–0.9 = very high, >0.9 = practically perfect) and CV magnitude (>10% = poor, 5–10% = moderate, <5% = good) was assessed using the criteria recently presented in the literature [20]. Statistical analyses associated with the least-products regression and ICC were performed using the Statistical Package for the Social Sciences software (version 25; SPSS Inc., Chicago, IL, USA). The SEM and CV were calculated in a spreadsheet following the guidelines presented by Batterham and George [19].

## 3. Results

**Figure 2:**
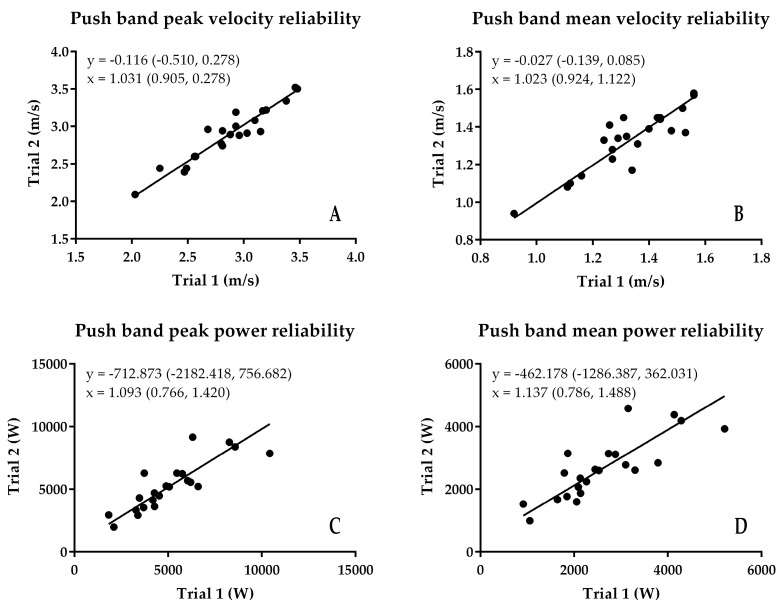
Results of the assessment of Push Band reliability using least products regression. (**A**) peak velocity; (**B**) mean velocity; (**C**) peak power; (**D**) mean power.

**Figure 3:**
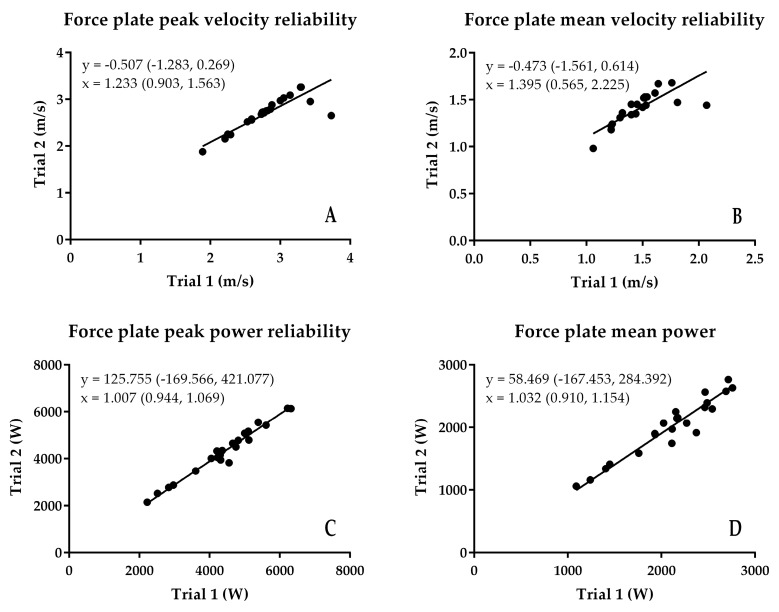
Results of the assessment of force platform reliability using least products regression. (**A**) peak velocity; (**B**) mean velocity; (**C**) peak power; (**D**) mean power.

**Figure 4:**
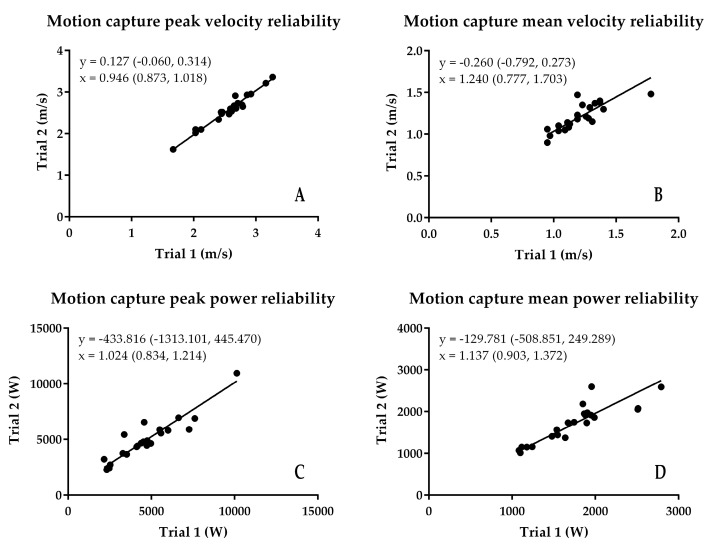
Results of the assessment of motion capture reliability using least products regression. (**A**) peak velocity; (**B**) mean velocity; (**C**) peak power; (**D**) mean power.

**Figure 5:**
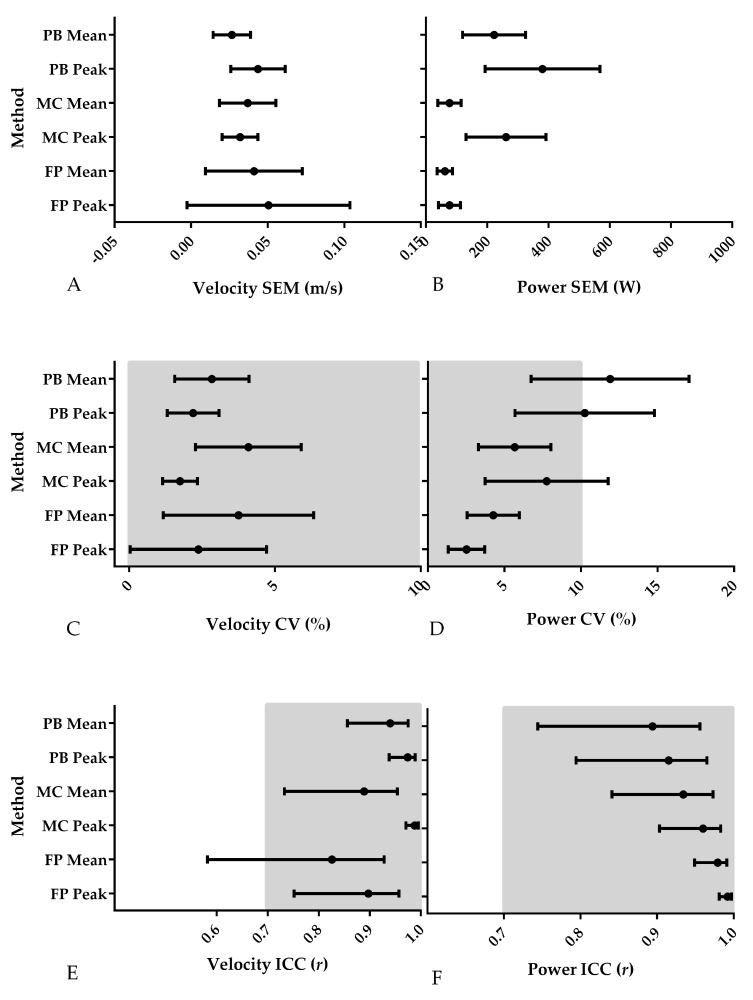
Results of the assessment of the reliability of the three different methods using SEM, CV and ICC; greyed out sections represent acceptable absolute and relative CV and ICC values; PB = Push Band, MC = motion capture, FP = force plate. (**A**) Velocity SEM (m/s); (**B**) Power SEM (W); (**C**) Velocity CV (%); (**D**) Power CV (%); (**E**) Velocity ICC (*r*); (**F**) Power ICC (*r*).

The results of the reliability analysis are presented in Figure 2, Figure 3, Figure 4 and Figure 5. Although there were some subtle variations between the first and second trial values, because the 95% confidence interval of the intercept passed through zero, there was no fixed bias present in any of the variables for any of the measurement techniques. Additionally, because the 95% confidence interval of the slope passed through 1, there was no proportional bias present for any of the variables for any of the measurement techniques. Additionally, while the SEM of the peak and mean velocity and power from the different methods was relatively small (Figure 5A,B), and absolute (Figure 5C) and relative (Figure 5E) peak and mean velocity reliability were good and high to very high, and relative peak and mean power reliability were high to very high (Figure 5F), absolute Push Band peak and mean power exceeded the CV 10% cut-off threshold for acceptable absolute reliability.

**Figure 6:**
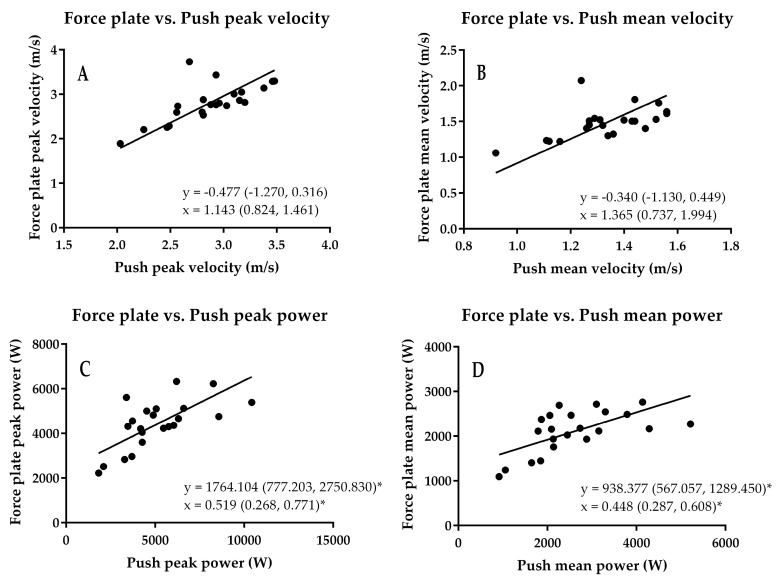
Results of the force plate vs. Push Band comparison. The * shows where fixed and proportional bias occurred. (**A**) peak velocity; (**B**) mean velocity; (**C**) peak power; (**D**) mean power.

**Figure 7:**
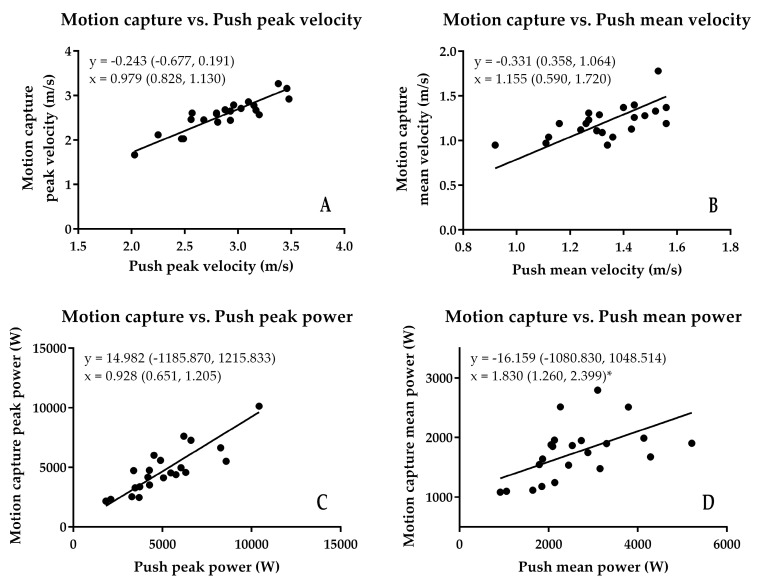
Results of the motion capture vs. Push Band comparison. The * shows where proportional bias occurred. (**A**) peak velocity; (**B**) mean velocity; (**C**) peak power; (**D**) mean power.

**Figure 8:**
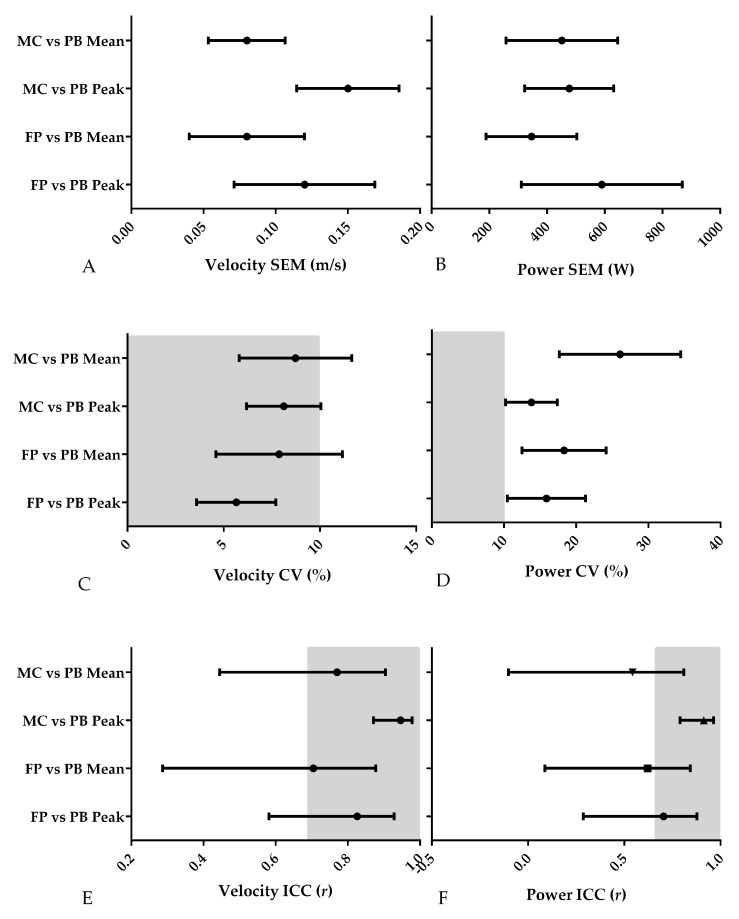
Results of the assessment of the validity of the three different methods using SEM, CV and ICC; greyed out sections represent acceptable absolute and relative CV and ICC values; PB = Push Band, MC = motion capture, FP = force plate. (**A**) Velocity SEM (m/s); (**B**) Power SEM (W); (**C**) Velocity CV (%); (**D**) Power CV (%); (**E**) Velocity ICC (*r*); (**F**) Power ICC (*r*).

The results of the method comparison are shown in Table 1 and Figure 6, Figure 7 and Figure 8. The results of the comparison between the Push Band and force platform peak velocity showed that while the Push Band tended to overestimate (0.477 m/s), there was no fixed or proportional bias. The SEM was equivalent to 4.2% of peak force plate velocity (Figure 8A), while the CV were moderate (5.7%), and the ICC was very high (*r* = 0.826). These findings also applied to mean velocity—the Push Band overestimated mean velocity by 0.340 m/s (Figure 6). Additionally, the SEM was equivalent to 5.4% of mean force plate velocity, while the CV was moderate (5.4%), and the ICC was high (*r* = 0.704). However, when peak power obtained from the Push Band and force platform were compared, the results showed that the Push Band underestimated by 1764 W and that both fixed and proportional bias were present (Figure 6). Additionally, the SEM was equivalent to 13.3% of peak force plate power, while the CV exceeded the 10% cut-off threshold (15.9%), but the ICC was high (*r* = 0.704). This underestimation extended to the comparison between mean power obtained by these two methods (938 W) and both fixed and proportional bias were present here too (Figure 6). Additionally, the SEM was equivalent to 16.4% of the mean force plate power, while the CV exceeded the 10% cut-off threshold (18.3%), and the ICC was high (*r* = 0.621).

When the Push Band was compared to motion capture, the Push Band overestimated both peak (0.243 m/s) and mean (0.331 m/s) velocity, but did not demonstrate fixed or proportional bias (Figure 7). The SEM was equivalent to 6.0% and 6.5% of motion capture peak and mean velocity. Additionally, their respective CV (peak = 8.1%, mean = 8.7%) and ICC (peak *r* = 0.946, mean *r* = 0.770) were moderate and high to very high (Figure 8). While the Push Band underestimated peak power by around 15 W, there was no fixed or proportional bias (Figure 7). While the Push Band appeared to overestimate mean power (16 W), there was no fixed bias. However, there was proportional bias (Figure 7) so that differences would increase proportionally to the magnitude of the Push Band output. The SEM were 10.0% and 25.8% for peak and mean power, respectively. While the peak velocity CV exceeded the 10% cut-off threshold (13.8%), the ICC was very high (*r* = 0.913). The mean power CV also exceeded the 10% cut-off threshold (26.1%), and the ICC was at the lower end of high (*r* = 0.543) (Figure 8).

## 4. Discussion

The aim of this study was to assess the validity of the peak and mean velocity and power obtained from the belt-worn Push Band and equivalent data derived from three-dimensional motion capture, and vertical ground reaction force during CMJ. Based on literature that has assessed the validity of the Push Band during jumping and different resistance exercises [4,5,6,7,8], it was hypothesized that the Push Band would not agree with laboratory-based gold standard methods. In general, while the Push Band tended to overestimate, it was suitable for recording peak and mean velocity. However, although peak power from the Push Band and motion capture agreed, mean power demonstrated proportional bias (Figure 7).

Additionally, peak and mean power obtained from the force plate and Push Band did not agree, with results demonstrating fixed and proportional bias (Figure 6). With regard to velocity, all the methods used in the present study demonstrated acceptable agreement. Additionally, it is important to note that velocity derived from the force plate represents the center of mass, whereas velocity derived from motion capture represent the velocity of the Push Band. This is important, because while it has been shown that there are no differences between the two during unloaded jumping, adding load can cause meaningful differences (extrapolated from data presented in their tables: 13%, effect size = 0.937 with 10% of back squat one repetition maximum) [21]. It should also be considered that peak velocity can be used to estimate jump height. However, unpublished data from the first author’s laboratory show that take-off tends to occur around 27 ms after peak velocity and that using velocity this way could yield differences of 0.162 m/s (6% of the peak velocity in this example). This could in turn lead to jump height overestimations of 0.04 m (13%). Ripley and McMahon [8] found that the Push Band overestimated peak velocity by 12%. This supports the difference of 17% we found when comparing the force plate and Push Band, although it should also be noted that the mean (SD) peak velocity recorded in the present study was 2.802 (0.430) m/s, which is 20% larger than peak velocity recorded by Ripley and McMahon [8]. This suggests that athlete standard may influence differences in peak velocity, although it could also be a consequence of the Push Band placement. In the present study the Push Band was worn on a belt, while Ripley and McMahon [8] placed the Push Band on the forearm in their study.

With regard to mean velocity, nothing is known about differences between the Push Band and laboratory-based gold standard methods during the CMJ. However, while Sato et al. [7] did not report descriptive data, they did report that there were no significant differences between mean velocity recorded from the Push Band (forearm-worn) and motion capture during the shoulder press and biceps curl exercises. Additionally, Banyard et al. [6] reported low agreement between the Push Band and other field-based methods during back squat with loads at or above 60% of one repetition maximum. Additionally, while Balsalobre-Fernández et al. [5] reported ‘good agreement’ between a linear position transducer and the Push Band during the back squat exercise performed in a Smith Machine, the Push Band overestimated mean velocity by 12.5%. Additionally, estimates from the 95% limits of agreement graphs presented by this group showed that there was a systematic bias of approximately 10%. While this may not be an issue because of the high reliability they reported, it is also important to note that all data (taken from back squats with different loads) was pooled for their statistical analysis, and this can influence the results of such tests. When comparing peak and mean velocity from the Push Band and motion capture, there were no fixed or proportional bias. This means that if practitioners remember that the Push Band can overestimate peak velocity by 9% and mean velocity by 27%, it could provide a useful device to assess velocity capacity during CMJ.

With regard to peak power, the results of the reliability analysis suggest that the three methods provided acceptable reliability. To the authors’ knowledge, only one study has compared the peak power determined by the Push Band and force plate during jumping [8]. It should be noted that the subjects wore the Push Band on the forearm during this study making comparison with the results of the present study difficult. However, as with peak velocity, their results agree with the results of the present study in that the Push Band overestimates peak power by around 7% when compared to the force plate, which is less than the 40% overestimation found in the present study. In addition to this large difference, results of the least-products regression showed that there was fixed and proportional bias. Therefore, the Push Band should not be used to measure CMJ peak power. When compared to motion capture peak power, the difference was considerably smaller (0.3%), but did not demonstrate fixed or proportional bias. This is likely to be a consequence of the fact that velocity should, in theory at least, reflect that of the Push Band because the marker that was tracked to obtain velocity was affixed to the Push Band. However, it should be noted that while there was no fixed or proportional bias for peak or mean velocity, the Push Band did overestimate these variables by 9 and 27%, respectively. To calculate force from the motion capture method marker displacement was differentiated twice to obtain the acceleration before force could be calculated and multiplied by the velocity of the marker. Therefore, it seems that the force derived from the motion capture method must have been less than that of the Push Band to balance out the force-velocity components of the power calculation. Practitioners and researchers should bear this in mind if they plan to use the Push Band.

Similarly to the points raised for mean velocity, very little is known about the validity of mean power determined with the Push Band during CMJ. The results of the comparison between the mean power determined with the Push Band and the mean power derived from the force plate method indicate that the Push Band overestimated mean power by 45% and demonstrated both fixed and proportional bias. While the systematic bias was considerably lower when the Push Band was compared to the motion capture method (3% compared to 45%), it still demonstrated proportional bias. This suggests that regardless of the laboratory-based system that is used to validate Push Band mean power the agreement between the systems is unacceptable. As such, practitioners should avoid using the Push Band to assess CMJ mean power. Banyard et al. [6] reported that Push Band mean power did not meet their criteria for high validity during back squat exercise, with loads equal to 40% of one repetition maximum and above when compared to their four linear position transducer system. However, these findings should be considered with caution when compared to the results of the present study because the laboratory-based measurement methodology considered different points. For example, the laboratory-based methods used in the present study considered center of mass (force plate) and Push Band positional (motion capture) mean power. Regardless, the results of the present study indicate that the Push Band should not be used to measure CMJ mean power. 

## 5. Conclusions

The results of this study show that while the Push Band overestimates peak (9–17%) and mean (23–27%) velocity, it does not demonstrate fixed or proportional bias. Therefore, if these differences are considered, the Push Band can be used to reliably record CMJ peak and mean velocity. However, while the Push Band and motion capture peak powers agree, the Push Band and force plate peak powers do not. Additionally, mean power demonstrated fixed and proportional bias when compared to both the force plate and motion capture methods so cannot be considered valid.

## Figures and Tables

**Table 1:** Mean (SD) force plate and Push Band peak and mean velocity and power, the mean (SD) and 95% confidence interval of the differences between them.

Dependent Variable	Mean (SD) Force Plate	Mean (SD) Push Band	Mean (SD) Difference	95% Confidence Interval
Peak velocity (m/s)	2.802 (0.430)	2.870 (0.377)	0.068 (0.312)	−0.070, 0.206
Mean velocity (m/s)	1.481 (0.221)	1.334 (0.163)	−0.147 (0.186)	−0.230, −0.065 *
Peak power (W)	4418 (1087)	5109 (2092)	691 (1593)	−15, 1397
Mean power (W)	2106 (475)	2585 (1082)	502 (862)	120, 885 *

Note: SD = standard deviation, *d* = effect size, m/s = meters per second, W = watts, * = significant difference.
